# Case of an Excrescence in the Urethra of a Female Patient Successfully Treated

**Published:** 1786

**Authors:** J. C. Jenner

**Affiliations:** Surgeon, at Painswick, in Gloucestershire


IX. *Caſe of an Excreſcence in the Urethra of a female Patient ſucceſsfully treated. By Mr.* J. C. Jenner, *Surgeon, at Painſwick, in Glouceſterſhire.*

MRS. B. aged thirty, began, about fifteen years ago, ſoon after the firſt appearance of the menſes, to be afflicted with an almoſt-conſtant irritation to void her urine. As her menſes were irregular, her complaint, it was ſuppoſed, might be owing to that circumſtance, and ſhe took a variety of medicines, but without any good effect, as the irritation increaſed to ſuch a degree, that ſhe was obliged to void her urine every ten or fifteen minutes. If ſhe omitted to do this the moment the irritation returned, ſhe never failed to experience great pain. This ſenſe of irritation even waked her in the night, and ſhe ſeldom ſlept longer than half an hour at a time.

The urine was no ſooner evacuated, than ſhe felt a ſevere pain, which commonly laſted about two minutes. After this ſhe remained eaſy about ten minutes, and then the irritation returned in the ſame manner as before.

Sometimes the urine flowed freely; at other times it came away only by drops; and now and then it ſtopped ſuddenly. She had frequently an inclination to go to ſtool, but was, in general, coſtive.

In this manner her complaint continued, with little or no variation, till about four years ago, when, after voiding a few drops of urine, with much pain, ſhe diſcharged about four ounces of blood. Her caſe was now pronounced to be the ſtone in the bladder by two medical gentlemen whom ſhe conſulted; and ſhe herſelf being ſatisfied that this opinion was well founded, and determined not to ſubmit to an operation, had recourſe to different remedies; but, finding finding no good effect from them, had, for a considerable time before she applied to me, taken only an infusion of sena occasionally, to procure a few stools, and this, she thought, relieved her more than any other medicine had done.

In this painful situation she had lived fifteen years, when, about four months ago, she related her case to me, but without any hope of relief — I requested leave to examine the seat of the complaint; and this being granted, I found a fungous excrescence filling up the orifice of the meatus urinarius. I immediately passed a ligature round it, and, by means of a pair of small sciffars, removed it at its origin, which was about a quarter of an inch within the urethra. The day following I introduced a bougie into the urethra, and suffered it to remain there an hour. This practice was repeated every day for a fortnight. There was no stone in the bladder; and the patient is now free from pain and irritation, and can retain her urine eight or ten hours together.

*Painswick,*

April 5, 1786.

